# Exploring Male Body Image: A Scoping Review of Measurement Approaches and Mental Health Implications

**DOI:** 10.3390/ijerph22060834

**Published:** 2025-05-26

**Authors:** Emily Pomichter, Antonio Cepeda-Benito, Shahrzad Ahmadkaraji, John P. DePalma

**Affiliations:** Department of Psychological Science, University of Vermont, Burlington, VT 05405, USA; emily.pomichter@uvm.edu (E.P.); shahrzad.ahmadkaraji@uvm.edu (S.A.); john.p.depalma@uvm.edu (J.P.D.)

**Keywords:** body image (BI), male body image, muscularity-oriented measures, thinness-oriented measures, mental health outcomes

## Abstract

Background: Body image (BI) concerns in men—spanning thinness, muscularity, and other related constructs—are increasingly being recognized for their links to depression, anxiety, and self-esteem, yet measurement approaches remain unevenly aligned. Objectives: Our objective was to map the landscape of BI instruments deployed with cisgender men and women and to examine whether thinness-, muscularity-, and non-specific self-image satisfaction measures differentially relate to key mental health outcomes. Eligibility Criteria: Our eligibility criteria were peer-reviewed, quantitative studies published in English between January 2011 and December 2021. We only included studies with samples ≥30 cisgender men, psychometrically validated BI scales, results reported separately by gender, and U.S.-based investigations. Sources of Evidence: The source of evidence was a systematic search of PsycINFO. Charting Methods: Two reviewers double-screened titles/abstracts and full texts in EPPI-Reviewer. Data on measure orientation; validation sample characteristics; and effect sizes relating BI scores to depression, anxiety, and self-esteem were extracted. The effect sizes were transformed to *r* and averaged across the studies. Results: Of the 1178 records identified, 191 U.S. studies met the inclusion criteria. Fifty-five distinct BI instruments were used; twelve appeared in six or more studies and were classified as thinness-oriented, muscularity-oriented, or non-specific. The unweighted average correlations with depression ranged from *r* = 0.23 (muscularity) to 0.34 (non-specific), with anxiety at *r* = 0.16–0.25 and self-esteem at *r* = 0.20–0.57. The male samples showed greater heterogeneity of effect sizes, likely reflecting the sampling variability and the multifaceted nature of men’s BI concerns. Conclusions: This review confirms critical gaps in male BI measurement and interpretation. Although thinness- and muscularity-oriented scales each capture facets of men’s BI, exclusive reliance on thinness measures risks overlooking leanness- and musculature-focused pathology. The greater variability of BI–mental health associations in men than women underscore the need for a diverse measurement toolkit that, at minimum, assesses thinness, muscularity, and other appearance-related constructs.

## 1. Introduction

By age 40 in the United States, approximately 14.3% of men and 19.7% of women are diagnosed with one of the three most common eating disorders (EDs): anorexia nervosa (AN), bulimia nervosa (BN), or binge eating disorder (BED) [1]. These statistics are particularly concerning, in part, because of the high comorbidities and elevated mortality rates associated with EDs. AN increases mortality rates sixfold, while BN doubles the risk of death [2]. In addition to mortality, over 94% of women with EDs experience co-occurring psychiatric conditions such as mood and anxiety disorders, while many also struggle with substance use, suicidal behaviors, or personality disorders [3,4,5].

Body image (BI) refers to an individual’s subjective perception of their physical appearance, along with the associated emotional responses, beliefs, and behaviors [6,7]. Concerns related to body image—such as negative evaluations of body size, shape, or weight—have consistently emerged as key predictors of ED symptoms [8,9,10]. Behaviors such as restrictive dieting, excessive exercising, and non-prescribed laxative use are often motivated by a desire to achieve an idealized body image [11,12]. BI concerns are also strongly linked to poor mental health outcomes, including low self-esteem [13,14], depression [15,16], and anxiety [17,18].

Much of the existing research on BI and its relationships with EDs and mental health has focused on women, for whom BI concerns are widespread in affluent Western societies [19]. Western cultural norms have historically emphasized physical beauty in women, promoting an unrealistic thin ideal [20]. Cultivation theory posits that prolonged exposure to media depicting idealized beauty shapes individual attitudes and values, encouraging women to internalize the thin ideal and become dissatisfied with their bodies [21]. Over decades, this ideal has evolved to incorporate a toned, ultra-fit aesthetic, further reinforcing BI concerns among the women exposed to such imagery [22,23].

As the ED prevalence among men has grown, scholarly attention has increasingly turned toward male BI concerns. Between 2000 and 2022, the number of peer-reviewed articles on male BI concerns in the PsycINFO database increased from 44 to 468, reflecting heightened interest in understanding this phenomenon. Male BI concerns are similarly driven by a desire for an idealized body shape, but its manifestations often differ from those in women. Many men express a desire for muscularity, thinness, or a combination of the two [24,25]. Studies using contour–silhouette scales have revealed that men are almost evenly split between those wanting to gain and those wanting to lose body size, while cross-cultural research has shown a preference for larger physiques among men compared to women’s preference for thinness [26,27,28].

Cultivation theory also applies to male BI concerns, as media depictions increasingly idealize muscular, V-shaped physiques as the standard for male beauty and masculinity [29]. Leit et al. [30] documented increased muscle mass and decreased body fat trends among male Playgirl centerfold models, while Pope et al. [31] found similar trends in male action figures. These portrayals reinforce societal preferences for dense, muscular bodies, which have become the dominant ideal for men [32].

Clinically diagnosed eating disorders such as anorexia nervosa (AN) and bulimia nervosa (BN) are less prevalent in men than women [33]. By contrast, muscle dysmorphia—a form of body-image disturbance characterized by an excessive drive for muscularity—has appeared almost exclusively in male samples [31,34]. These distinctions matter because drive-for-thinness scales map directly onto the AN/BN diagnostic criteria (e.g., fear of weight gain), whereas drive-for-muscularity instruments assess the core pathology of muscle dysmorphia (e.g., compulsive weight training).

### 1.1. Prior Reviews of Body Image Measures

Building on the foundational understanding of BI, examining how previous reviews have synthesized measures and identified limitations has become essential. When men’s body image emerged as a significant area of interest, researchers rapidly developed self-report questionnaires to measure this construct reliably. While these tools enable researchers to investigate critical questions about BI, the resulting proliferation of measures has created challenges. Differences in how various instruments assess aspects of BI can lead to inconsistencies across studies, with some measures varying in reliability and validity. To address these challenges, researchers periodically synthesize findings on BI measures, comparing their qualities and psychometric properties.

Previous reviews of BI assessment instruments often focused on specific populations (e.g., cancer patients [35]) or techniques, such as silhouette-drawing scales [36]. While such reviews have provided valuable insights within narrow contexts, they have failed to address the broader diversity of BI measures. This narrow scope limits our understanding of how various measures perform across different populations or capture distinct facets of BI, leaving significant gaps in the literature regarding male-specific or general BI assessment. 

Kling et al. [37] conducted the most systematic review of BI measures to date, focusing on three years (August 2011 to August 2014), to provide a snapshot of contemporary tools. The eight measures chosen by Kling et al. [37] were:

1. The Body Dissatisfaction Scale of the Eating Disorder Inventory (EDI BD);
2. The Drive for Thinness subscale of the EDI;
3. The Body Shape Questionnaire (BSQ);
4. The Shape and Weight Concern subscales of the Eating Disorder Examination Questionnaire (EDE Q);
5. The Body Areas Satisfaction Scale (BASS);
6. The Contour Drawing Rating Scale (CDRS);
7. The Body Image Ideals Questionnaire (BIQ);
8. The Body Image Satisfaction Scale (BISS).

All eight were originally validated in predominantly female samples and focus on smaller overall body size or global satisfaction—essentially assessing “thinness” or general contentment with appearance. However, male body image ideals center on leanness—a low fat-to-high muscle ratio—and musculature rather than thinness. Consequently, exclusively using female-focused tools can misrepresent men’s core concerns and risk misleading conclusions, which has motivated the development and application of drive-for-muscularity and other male-oriented measures.

### 1.2. The Current Review

We note that Kling et al. [37] conducted the most systematic review of BI measures, focusing on three years (2011–2014) to provide a snapshot of contemporary tools. This timeframe was chosen to ensure a manageable scope and reflect measures actively used in recent research. However, this decade-old study excludes by necessity those developments in BI assessment that have emerged since 2014.

Researchers often criticize an assumed reliance on thinness-oriented measures to assess male BI by citing that men generally report more drive for muscularity [38] and less concern with thinness [39]. However, no research has systematically examined how drive-for-thinness and drive-for-muscularity measures have been applied to assess male BI. Thus, this review aims to describe and synthesize the diversity of the measures currently used to assess BI in men by conducting a scoping review of studies measuring male BI and then categorizing the measures used into thinness-oriented, muscularity-oriented, and general appearance and non-specific (neither thinness- nor muscularity-oriented) BI measures. This simple classification process will allow us to assess the extent to which thinness-oriented measures are used to assess BI in men.

A second aim of this study is to explore the extent to which the practice of using thinness-oriented measures to assess male BI may lead to arriving at erroneous or misleading conclusions. For example, there is considerable research investigating the relationships between BI concerns and mental health outcomes, particularly self-esteem, anxiety, and depression. This research has consistently found that BI concerns are associated with and are likely a precursor of poor mental health, including low self-esteem [13,14], depression [15,16], and anxiety [17,18]. Barnes et al. [17] systematically reviewed studies published between 2008 and 2018 that examined the relationships between BI concerns and anxiety or depression in men. These authors [17] reported that although many studies found that BI concerns correlated positively with depression and anxiety, these effects did not replicate in about one-fourth of the studies. We speculate that the inconsistent associations between BI concerns and mental health outcomes could be a function of the types of BI measures used in the studies. We propose that classifying results by type of BI measure may clarify the potential consequences of using thinness-oriented measures to assess male BI.

### 1.3. Research Questions

To address the gaps in male body-image assessment and its mental-health implications, we pose the following research questions:

RQ1: Which types of body image (BI) measures—thinness-oriented, muscularity-oriented, or general/non-specific—have been most frequently used in studies conducted with or that include men?

RQ2: How do these three measurement orientations differentially relate to key mental health outcomes (depression, anxiety, self-esteem) in male and female comparable samples?

RQ3: Does exclusive reliance on thinness-oriented BI instruments risk producing misleading conclusions in men, thereby justifying the development and use of muscularity-oriented measures?

### 1.4. Scoping Review Purpose (PCC)

This review examines (P) cisgender male participants; (C) body image (BI) measures—thinness, muscularity, and general orientations—and their relationships to depression, anxiety, and self-esteem within the (C) context of peer-reviewed quantitative U.S. studies published in English from 2011 to 2021.

## 2. Methods

### 2.1. Research Framework

As Arksey and O’Malley [40] outlined, this study employed state-of-the-art criteria for scoping reviews. This framework includes five steps: identifying the research question; identifying relevant studies; selecting studies; charting the data; and collating, summarizing, and reporting the results.

### 2.2. Identification and Selection of Studies

**Figure 1:**
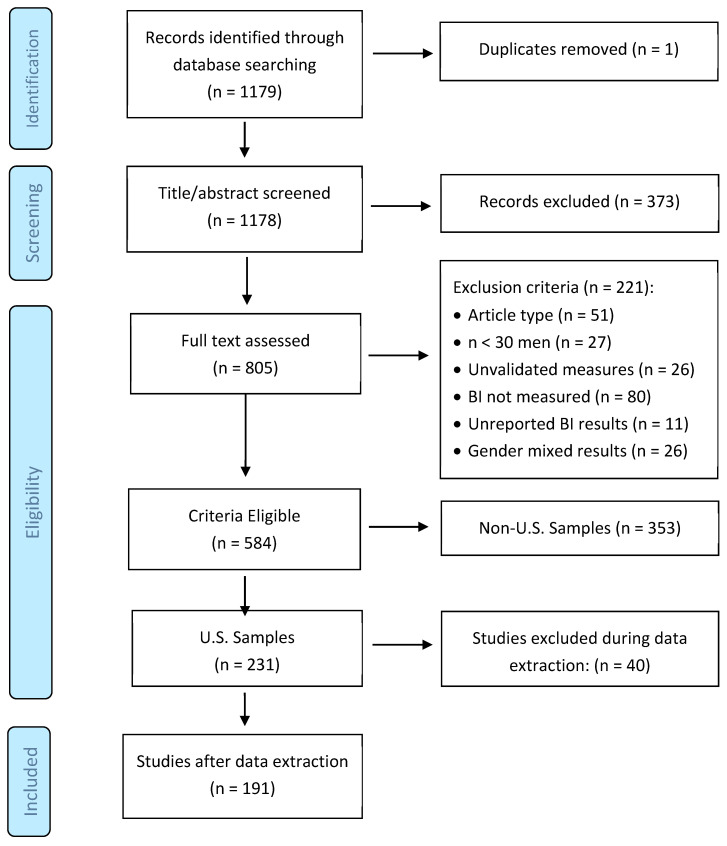
Identification and selection of studies.

We searched for research on body image that included male participants through PsycINFO: *noft(body image OR body dissatisfaction OR muscularity) AND noft(men OR man)*. The search, limited to peer-reviewed articles published in English between 1 January 2011 and 31 December 2021, yielded 1179 studies (see Figure 1).

We selected PsycINFO as our sole database because it is the most comprehensive index of peer-reviewed journals in psychology and eating-disorder research.

### 2.3. Screening Process

The titles and abstracts were double-screened using EPPI-Reviewer software by two undergraduate research assistants. The assistants were trained to use EPPI-Reviewer and were required to achieve at least 80% agreement in practice trials before starting the screening process. In cases of disagreement, a third reviewer resolved disputes. The agreement rate during the review of the titles and abstracts was 86%, and 805 studies were selected for full-text review. The agreement rate was 83% in the full-text screening phase, and 584 studies met the inclusion criteria. The inclusion criteria looked for empirical or quantitative studies, included cisgender males (n > 29 cisgender males), used psychometrically validated BI measures, presented data segregated by gender, and reported BI results. To increase the focus and reduce the heterogeneity and scope of the sample, we further limited the scope to U.S.-based investigations (n = 231).

**Exclusion Criteria**: Of the 805 full-text articles assessed, 221 were excluded for the following reasons (Figure 1):

- Non-empirical, non-quantitative, or qualitative publications, or conference abstracts (n = 51);
- Sample size of fewer than 30 cisgender men (n = 27);
- Use of unvalidated body image measures (n = 26);
- Body image not assessed (n = 80);
- Results not disaggregated by gender (n = 26);
- Insufficient quantitative results for effect-size extraction (n = 11);

In addition, 353 studies were excluded at this stage because they were conducted outside the United States, yielding a sample of 231 U.S.-based investigations. An additional 40 studies provided insufficient detail during data extraction and were eliminated. Thus, the final sample size included 191 studies. 

Although over 60% of the articles identified in the full-text screening were conducted outside the United States, we elected to limit our review to the U.S.-based samples. This decision was made to minimize the cultural and measurement heterogeneity—such as differences in body-image norms, translation of instruments, and sociocultural ideals—that would otherwise have substantially expanded the scope of our synthesis beyond its intended focus on thinness- vs. muscularity-oriented measures in a single cultural context.

### 2.4. Classification of Measures

By consensus, this study’s authors classified the measures into thinness-oriented, muscularity-oriented, and general/unspecific. The thinness-oriented measures assessed one or a combination of the following: perceiving weight gain as undesirable, internalizing the thinness ideal of beauty, or behaviors aimed at losing or not gaining weight. The drive for muscularity measures assessed any of the following: a preoccupation with not gaining enough weight or strength, a desire to increase body size, and behaviors aimed at being muscular. These scales were typically developed and validated with male samples. We classified as unspecific or general measures those that primarily assessed satisfaction with physical appearance or body functionality but did not depend substantively on acquiring or maintaining a thin or muscular body shape and size.

**Measure Inclusion Threshold**: To identify the most substantively studied instruments, we included only those used in six or more studies—a cutoff representing the top quartile of measure-use frequency (see Table 1). This pragmatic threshold balances depth (focusing on widely applied scales) with feasibility and aligns with precedents in prior scoping and psychometric reviews of body-image instruments [35,36].

Additional data extraction was then conducted for all studies that assessed the relationships of BI concerns with depression, anxiety, or self-esteem. The studies that examined the relationships between BI and at least one measure of depression, anxiety, or self-esteem were identified. We then extracted the statistics that reported the relationships between the BI scores and any of the three outcome measures. This process was carried out for each male and female sample and then summarized by transforming all effect sizes to *r* and simply averaging them across the studies.

We elected to focus on depression, anxiety, and self-esteem—outcomes that are comorbid with EDs but distinct from diagnostic criteria—because including ED or disordered-eating endpoints would have conflated our predictors (drive-for-thinness and drive-for-muscularity measures) with clinical diagnostic features. Our second aim was to test whether reliance on thinness-oriented tools risks misleading conclusions for men and how they compare to women—questions not resolved by examining ED diagnoses, which, by definition, correlate with their respective drive-for-thinness or drive-for-muscularity scales.

## 3. Results

### 3.1. Study Characteristics

The sample of studies included 18 validation articles describing the development and psychometric validation of new measures of body image and 173 non-validation articles that used body image measures in their research. The non-validation studies varied geographically; 37 were in the Northeast, 35 in the Southeast, 11 in the West, 14 in the Southwest, and 37 in the Midwest (39 studies did not specify the geographic locations of their samples). The data for 40 studies were collected online, while 133 involved in-person data collection. Of the in-person samples, 77 consisted of university students and 55 of community samples. Several studies recruited from specific identity-based populations, including sexual-minority men (n = 32), athletes (n = 3), populations with histories of disordered eating (n = 5), men with HIV (n = 3), and ethnic/racial minorities (n = 10). Regarding the gender composition of the samples, 97 studies included both men and women, 76 studies included men only, and one study included gender-non-binary individuals.

### 3.2. Relative Popularity of Thinness-, Muscularity-, and Non-Specific Measures

Fifty-five different measures of body image were utilized across the 173 non-validation studies. Of the 55 measures, 14 were muscularity-oriented, 22 were thinness-oriented, 2 assessed body image using both muscularity- and thinness-oriented questions, and 17 assessed were non-specific measures.

Of the 55 measures, only 12 were used in at least six different studies, which means that most measures were seldom and rarely used across the different studies. The muscularity- and thinness-oriented measures were used most frequently—108 and 104 times, respectively—and the non-specific measures were used 81 times.

Table 1 summarizes the characteristics of the 12 measures utilized in at least six of the studies. These measures are categorized into three orientations: thinness-oriented, muscularity-oriented, and non-specific. For each measure, this table provides information on validation samples (including gender, age range, and population) as well as reliability (e.g., internal consistency and test–retest reliability) and validity (e.g., criterion, convergent, or construct validity). Additionally, this table highlights the frequencies of their use in studies with at least 30 male participants, offering insights into each one’s relative popularity and applicability.

The thinness-oriented measures were validated on predominantly female samples, although their application in male populations has grown. For example, Gardner et al.’s Body Dissatisfaction Scale [41], the most frequently used thinness-oriented measure, was validated on females aged 20–22 in clinical and college settings. Similarly, other measures, such as Fairburn and Beglin’s Concerns, Shape, and Weight Scales [42] and McKinley and Hyde’s Objectified Body Consciousness Scales [43], were validated using female samples spanning ages 16–39, primarily from community or college populations. These measures demonstrate strong reliability, with internal consistency values ranging from 0.72 to 0.91, and robust validity supported by criterion and convergent evidence. While historically female-focused, these measures reflect the enduring relevance of thinness-related body image concerns for men, albeit to a lesser extent than muscularity concerns.

Muscularity-oriented measures reflect the growing emphasis on male-specific body image concerns, such as muscularity and strength. Unlike thinness-oriented measures, the validating samples for the muscularity-oriented measures included both men and women, with a stronger emphasis on the male participants. For example, the Drive for Muscularity Scale [24]—the most frequently applied measure in this category—was validated on both genders, primarily high school students aged 18–24. Other measures, such as Tylka et al.’s Male Body Attitudes Scales [25], focused exclusively on male samples aged 16–62 in college settings. These measures consistently showed high reliability, with internal consistency values reaching up to 0.94, but their validity was more variable, with construct-related correlations ranging from strong to nonsignificant, depending on the related construct. This variability highlights the importance of refining these tools to better capture the nuances of male muscularity-related body image concerns.

Non-specific measures provide broader assessments of body image and satisfaction without focusing on specific dimensions like thinness or muscularity. The validating samples for these measures included both genders, with wide age ranges and diverse populations. For instance, Brown et al.’s Multidimensional Body Self-Relations Questionnaire [49] was validated on males and females aged 15–87 from community settings, while Avalos et al.’s Body Appreciation Scale [50] focused on college-aged women and men aged 18–22. These measures exhibited high reliability, with internal consistency values of around 0.94, and strong validity, showing substantive associations with related constructs. However, their generalist nature may limit their ability to fully address the unique body image concerns specific to men, such as the drive for muscularity or thinness.

### 3.3. Research on Mental-Health Outcomes and Body Image

#### 3.3.1. Thinness-Oriented Body Image Measures

Twenty-three studies used thinness-oriented measures of body-image dissatisfaction and investigated its relationship with depression, anxiety, or self-esteem. Fifteen of these studies recruited gender-mixed samples and eight used only male participants. Two studies utilized the Body Esteem Scale (BES) [46], which has gendered versions such that the female version is thinness-oriented and the male version is muscularity-oriented.

The sample sizes of the 23 studies ranged from 70 to 2621, with the median sample size being 405. The cumulative number of the participants added up to 14,153, of which men (n = 6506) comprised almost half (46%) of the total. In terms of the racial and ethnic distribution of the overall sample, most participants were White (73.7%), followed by other or mixed-race (6.2%), Asian/Pacific Islander (5.8%), Hispanic (5.5%), and Black (5.5%).

Most studies were conducted in the Midwest (n = 8), and the remainder were distributed across the U.S. Northeast (n = 4), Southeast (n = 4), Southwest (n = 1), and West (n = 3); Hawaii (n = 1); and multiple locations (n = 2). Most studies used convenience samples that recruited college students (n = 14). The remaining studies used exclusively community participants (n = 5) or a combination of college students and community participants (n = 3), and one used a middle-school sample.

The findings across the 23 studies using thinness-oriented body image measures suggest that body image dissatisfaction was studied across gender-mixed and male samples and that men comprised a substantive number of the participants (close to half). These studies also reflect the predominance of White participants and college-based convenience samples, which obviates the lack of the generalizability of the BI research.

Depression: Many studies that examined BI in men using thinness-oriented measures also included measures of depression and calculated the effect sizes of the relationship between BI and depression (n = 14). Some studies included more than one measure of BI, and those that involved gender-mixed samples provided separate effect sizes for men and women. Thus, many studies provided multiple effect sizes describing the relationship between BI scores and depression. Combined, these 14 studies reported 37 effect sizes, with more reported for men (n = 22) than women (n = 15). The cumulative number of the participants across the 14 studies was rather large: 8388 (men = 3629; women = 4759). Overall, the average effect size of the relationship between high drive for thinness BI and depression was approximately equal for the men (*r* = 0.30) and the women (*r* = 0.32), but the range of the effect sizes was wider for the men (−0.10 to 0.90) than for the women (0.15 to 0.53).

Anxiety: Only five studies that examined BI in men using thinness-oriented measures also included measures of anxiety and calculated the effect sizes of the relationship between BI and anxiety. These five studies reported 17 effect sizes: 10 for men and 7 for women. The cumulative number of the participants across the five studies was considerable (n = 2170), with similar numbers of men (n = 1345) and women (n = 1413), although two of the five studies did not include women. Overall, the average effect size of the relationship between high drive for thinness BI and anxiety was slightly higher among the women (*r* = 0.25; range 0.06 to 0.76) as compared to the men (*r* = 0.16; range −0.39 to 0.71) but highly heterogeneous among both the men and women.

Self-Esteem: Of the 23 studies that examined BI in men using thinness-oriented measures, 12 included measures of self-esteem and calculated the effect sizes of the relationship between high drive for thinness BI and self-esteem. These 12 studies reported a total of 45 effect sizes, with about the same numbers calculated for men (24) and women (21). The cumulative number of the participants across the 14 studies was considerable (n = 6635), as were the respective numbers of the men (n = 3477) and women (n = 3228). Overall, the average effect size of the relationship between BI and self-esteem was smaller and more heterogeneous for the men (*r* = −0.25; range = −0.90 to 0.10) than for the women (*r* = −0.40; range = −0.53 to −0.15).

#### 3.3.2. Muscularity-Oriented Measures of Body Image

A total of 16 studies used muscularity-oriented measures of BI and mental-health outcomes. Of these, 10 used exclusively male samples and 6 used gender-mixed samples. The sample sizes of the 16 studies ranged from 100 to 1416, with the median sample size being 380. The cumulative number of the participants added up to 7849, of which men comprised the majority (69.3%). That is, there were 5440 male participants and 2409 female participants.

In terms of the racial and ethnic distribution of the overall sample, most participants were White (70.8%), followed by Hispanic (12.0%), other or mixed-race (9.0%), Asian/Pacific Islander (6.1%), and Black (4.5%). These studies distributed across the U.S. included the Midwest (n = 4), West (n = 4), Northeast (n = 2), Southeast (n = 2), and Southwest (n = 1) and multiple locations (n = 3). Most studies used convenience samples that recruited college students (n = 9). A few used a combination of college students and community participants (n = 3), and the rest used exclusively community participants (n = 4).

To summarize, the muscularity-oriented measures of BI were frequently used with the male participants, as demonstrated by the fact that 10 out of the 16 studies used exclusively male samples. Even in gender-mixed samples, men comprised a majority (69.3%) of the participants, emphasizing the focus on male BI concerns related to muscularity. However, the predominant use of White participants (70.8%) and reliance on convenience sampling, particularly among college students, again limits the generalization of this BI research. The geographic distribution was also somewhat skewed, with most studies concentrated in the Midwest and West regions of the U.S.

Depression: Of the 16 studies that examined BI in men using muscularity-oriented measures, 14 included measures of depression. These 14 studies reported a total of 28 effect sizes (24 for men and only 4 for women). The cumulative number of the participants across the 14 studies was considerable (n = 6996), with substantially more men (n = 5199) than women (n = 1797). Overall, the average effect size of the relationship between high drive for muscularity BI and depression seemed higher for the men (*r* = 0.23) than for the women (*r* = 0.19), with the range of the effect size being more heterogeneous for the men (0.03 to 0.70) than for the women (0.12 to 0.28).

Anxiety: Five studies that examined BI in men using muscularity-oriented measures calculated the effect sizes of the relationship between BI and anxiety. These five studies allowed for the calculation of a total of 10 effect sizes, only two of which were for women. The cumulative number of the participants across the five studies was considerable (n = 3645). There were 2744 men and only 901 women, all of whom were in the same study. Overall, the effect sizes of the relationship between drive for muscularity BI and anxiety were approximately equal between the men (average *r* = 0.23) and women (average *r* = 0.20), again with a much greater range of scores for the men (*r* range = 0.08 to 0.66) as compared to the women (*r* range = 0.18 to 0.22).

Self-Esteem: Six studies calculated 13 effect sizes (only three for women) of the relationship between BI and self-esteem using muscularity-oriented measures. The cumulative number of the participants across the six studies was 2078, with more men (*n* = 1296) than women (*n* = 782). Overall, the effect size of the relationship between drive for muscularity BI and self-esteem was higher for men (average *r* = −0.20) as compared to women (average *r* = −0.15), and there was a much greater range of scores for the men (*r* range = −0.46 to −0.02) as compared to the women (*r* range = −0.21 to 0.12).

#### 3.3.3. Non-Specific Measures of Body Image

Eighteen studies used BI concern measures that fit neither the thinness- nor the muscularity-oriented characterization. Of these, 13 studies recruited male participants exclusively, and five recruited gender-mixed samples. The sample sizes of the 18 studies ranged from 89 to 858, with a median sample size of 328. The cumulative number of the participants added up to 6592, with men making up the majority (84.6%). There were 5577 male participants and 1015 female participants.

In terms of the racial and ethnic distribution of the combined samples, most participants were White (66.7%), followed by Black (13.1%), Hispanic (10.3%), other or mixed-race (7.9%), and Asian/Pacific Islander (4.4%). The most significant number of studies were multi-site studies with locations across the U.S. (n = 6). The remaining studies were distributed across the country, including the Northeast (n = 4), Southwest (n = 3), Southeast (n = 2), Midwest (n = 2), and West (n = 1). Half of those studies used convenience samples that recruited college students (n = 9). Of the remaining samples, two used a combination of college students and community participants, six used exclusively community participants, and one used a sample of middle school students.

The studies using BI measures without a thinness or muscularity focus showed the strongest male representation (84.6%). Although the racial diversity slightly improved compared with the studies that used other types of BI measures, White participants still dominated (66.7%). Convenience sampling and limited regional diversity persisted, though a higher proportion of multi-site studies was included.

Depression: Twelve studies using non-specific measures of BI concerns reported 27 effect sizes (only four for women) for the relationship between BI and depression. The cumulative number of the participants across the 12 studies was considerable (n = 3799), with 89.6% being male (n = 3403) and with only 396 women. Overall, the average effect size of the relationship between BI concerns and depression was higher for the men (*r* = 0.34) than the women (*r* = 0.29), with a wider effect-size range for the men (0.09 to 0.75) than the women (0.20 to 0.38).

Anxiety: Five studies calculated a total of 18 effect sizes, seven of which were for women. The combined number of the participants for the five studies was 2270. There were 1940 men and only 330 women. The average effect sizes for the men (*r* = 0.24) and women (*r* = 0.27) were similar, but the variability of the effect sizes was greater for the men (range = 0.10 to 0.47) than the women (range = 0.20 to 0.36).

Self-Esteem: Seven studies that examined BI concerns in men using general body image measures also included measures of self-esteem and calculated the effect sizes of the relationship between BI and self-esteem. These seven studies allowed for the calculation of a total of 17 effect sizes; however, in one study, none of the effects were significant (for sexual-minority and straight men and women), and the effect size was not reported. Three of the remaining 13 effect sizes were for women. The cumulative number of the participants across the six studies was 2620, with more men (*n* = 1831) than women (*n* = 789). Overall, the effect size of the relationship between BI concerns and self-esteem was slightly higher for the women (average *r* = −0.57) as compared to the men (average *r* = −0.55), and there was a similar range of scores for the men (*r* range = −0.70 to −0.29) as compared to the women (*r* range = −0.70 to −0.35).

## 4. Discussion

This scoping review aimed to synthesize the literature on body image (BI) in men, focusing on the differential usage of thinness-oriented, muscularity-oriented, and non-specific BI measures as well as the associations between these measures and mental health outcomes such as depression, anxiety, and self-esteem. Our findings underscore the methodological trends and gaps in BI research, particularly in its application to male populations.

### 4.1. Study Characteristics and Measure Utilization

This analysis revealed that the research landscape on male BI remains constrained by several methodological limitations. Of the 173 non-validation studies included, the majority relied on convenience samples, with 77 studies recruiting university students and 55 utilizing community samples. Geographically, most studies were concentrated in the Midwest and Southeast regions of the United States, with limited representation from the Southwest and West. This geographic skew, combined with a reliance on predominantly White participants (over 70%), highlights the lack of diversity in BI research. Furthermore, the studies examining male BI disproportionately sampled gender-mixed populations (97 studies) compared to male-only populations (76 studies), and only one study included non-binary individuals.

In terms of measurement, 55 distinct BI measures were identified, yet only 12 were used consistently in at least six studies. Thinness-oriented measures were used 104 times, muscularity-oriented measures were used slightly more often (108 times), and non-specific measures were used less frequently (81 times). The thinness-oriented measures were predominantly validated on female samples, reflecting their historical origins in research on women’s BI concerns. In contrast, the muscularity-oriented measures were validated with male-focused samples, aligning with the male ideal of muscularity. The non-specific measures, though inclusive of broader BI constructs, lacked a clear focus on male-specific concerns like muscularity or thinness, potentially limiting their relevance in this context.

Despite their historical validation on female samples, this persistent reliance on thinness-oriented measures likely reflects the lag in the development and adoption of male-specific BI assessment tools. Muscularity-oriented measures, while more representative of male BI concerns, have remained relatively underutilized, particularly in mixed-gender studies. Nonetheless, these trends appear to have reversed course, as the most popular measure, used 51 times, was the Drive for Muscularity Scale [24], and another measure developed relatively recently, the Male Body Attitudes Scales [25], was also popular, used 23 times. By comparison, the most frequently used thinness-oriented measures were the Body Dissatisfaction Scale of the Eating Disorder Inventory [41], used 28 times, and the Concerns, Shape, and Weight Scales of the Eating Disorder Examination-Questionnaire [42], used 25 times.

### 4.2. Associations Between BI and Mental Health Outcomes

#### 4.2.1. Depression

Across all three BI measure types, BI was positively correlated with depression, though the magnitude of the association varied. The thinness-oriented measures yielded similar average correlations for men (r = 0.30) and women (r = 0.32), with a slightly wider range for the men (−0.10 to 0.90). The muscularity-oriented measures demonstrated smaller but still significant associations, with men showing higher average correlations (r = 0.23) than women (r = 0.19). The non-specific measures exhibited the strongest correlations for men (r = 0.34) compared to women (r = 0.29). These findings suggest that while thinness-oriented measures capture the BI–depression relationship similarly across genders, muscularity-oriented and non-specific concerns measures appear to be more sensitive to male than female-specific concerns. Nonetheless, the average correlation between depression and BI concerns in the men did not vary much as a function of the type of measure used (*r* 0.30 to 0.34). To the extent that these measures assess separate constructs, all seemed similarly associated with depression, and thus all similarly important and relevant to men’s mental health. This overall pattern aligns with the view that, compared to women, body image and physical appearance exert more variable influences on men’s mental health [43].

#### 4.2.2. Anxiety

The relationship between BI concerns and anxiety was highly variable, particularly for men. The thinness-oriented measures showed weaker average correlations for men (r = 0.16) compared to women (r = 0.25), with the men displaying a much wider range of effect sizes (−0.39 to 0.71). The muscularity-oriented measures produced more consistent correlations, with men and women showing similar averages (r = 0.23 and r = 0.20, respectively). The non-specific measures yielded comparable average correlations for men (r = 0.24) and women (r = 0.27), but with greater variability for the men.

In contrast to the findings related to depression, these findings suggest that thinness-oriented tools vs. muscular-oriented and non-specific measures underestimate or capture an aspect of BI that is less relevant to anxiety in men than women. However, temper this conclusion by acknowledging that it is based on analyzing relatively few effect sizes.

#### 4.2.3. Self-Esteem

Overall, the average effect size of the relationship between BI concerns and self-esteem was smaller and more heterogeneous for men (r = −0.25) than for women (r = −0.40). This pattern underscores that low self-esteem associations with BI concerns differ more across men’s samples than women’s. Conversely, the muscularity-oriented measures revealed stronger correlations for men (r = −0.20) compared to women (r = −0.15), underscoring the salience of muscularity in male self-esteem. The non-specific concerns measures showed similarly high correlations for both genders, with the women (r = −0.57) slightly higher than the men (r = −0.55). These findings suggest that self-esteem is more tightly associated with thinness than muscularity in women and the reverse in men. However, the most revealing finding was that self-esteem was most closely tied to general or non-specific physical appearance in both men and women.

## 5. Limitations

As this was a scoping review, we did not perform formal risk-of-bias or quality appraisal of the included studies [40]. This approach emphasizes mapping the breadth of evidence over critical evaluation, so readers should interpret the associations in light of possible variability in the study quality. Additionally, our reliance on unweighted average effect sizes and a single-database search may have limited the precision and comprehensiveness.

While our review focuses on the orientation of measures toward thinness versus muscularity to evaluate how these tools perform in male samples—and to examine whether exclusive reliance on thinness-oriented instruments may lead to misleading conclusions for men—it is important to recognize that these scales also span cognitive domains (e.g., negative body evaluations), affective domains (e.g., body shame), behavioral domains (e.g., weight-loss behaviors), and global versus body-part-specific formats (e.g., the Body Parts Satisfaction Scale). Alternative classifications that integrate constructs such as thin-ideal internalization and self-objectification alongside thinness and muscularity orientations may yield additional insights into men’s body-image concerns. We therefore recommend that future reviews explore these finer-grained taxonomies to deepen the understanding of body-image-related constructs in male populations.

By excluding direct ED/disordered-eating outcomes, we preserved a clear test of thinness- versus muscularity-oriented measures in male samples; however, we encourage future systematic reviews to investigate how these constructs relate to clinical ED and muscle dysmorphia diagnoses.

Our reliance on a single database (PsycINFO) may have omitted studies indexed elsewhere. Future reviews should consider searching for multiple databases (e.g., PubMed, Web of Science) to ensure maximal comprehensiveness.

Overall, the average effect sizes of the relationships of BI with depression, anxiety, and self-esteem were stronger for women compared to men but highly heterogeneous for both genders. This heterogeneity may partly reflect the sampling variability—several of the male-only and mixed-gender studies had relatively small Ns, which can inflate correlation estimates. Nevertheless, the data suggest that BI–mental health associations tend to be more variable in men than women.

Finally, our exclusive focus on U.S. samples may have constrained the generalizability of these findings to other cultural settings. We acknowledge that the inclusion of broader Western or international samples would have enhanced the external validity. We therefore recommend that future work directly compare U.S. and non-U.S. (or Western and non-Western) studies to determine the robustness and potential cultural specificity of thinness- and muscularity-oriented body image measures.

## 6. Conclusions

This review confirms critical gaps in the measurement and interpretation of male BI [38,39]. The review also highlights both the progress and ongoing challenges in understanding BI in men. Categorizing BI measures and examining their associations with mental health outcomes underscores the importance of using a wide spectrum of tools to capture the diverse aspects of BI concerns and their impact, particularly when investigating this phenomenon with men. The greater variability in the effect sizes for men across all mental health outcomes likely reflects the complex and diverse nature of male BI, which encompasses at least thinness, muscularity, and other concerns. In contrast, the review findings suggest that women’s BI appears more uniformly tied to thinness. Regardless, these findings underscore the need to measure BI in men and women using theoretically diverse measures, including, at a minimum, assessment of thinness, muscularity, and other appearance and body-related concerns.

## Figures and Tables

**Table 1:** Measures most frequently used to assess male body image or physical appearance satisfaction in men by type: thinness-oriented, muscularity-oriented, and non-specific.

Author and Measure (Studies’ n)		Validation Sample			Psychometrics
Author/Scale	(n)	Gen	Age	Pop	Reliability and Validity
Thinness-Oriented (n = 14 depression studies, avg *r* = 0.30 [men] and 0.32 [women]; n = 5 anxiety studies, avg *r* = 0.16 [men] and 0.25 [women]; n = 12 self-esteem studies, avg *r* = −0.25 [men] and −0.40 [women])					
Body Dissatisfaction Scale of the Eating Disorder Inventory [41]	(28)	Fem	20–22	Clin/Coll	Reliability: High internal consistency in both samples (~0.91). Validity: Criterion (significant mean differences between clinical and non-clinical subsamples) and convergent (high *r* with other body dissatisfaction measures [0.55 to 0.69]).
Concerns, Shape, and Weight Scales of the Eating Disorder Examination-Questionnaire [42]	(25)	Fem	16–35	Com/Clin	Reliability: Acceptable with high internal consistency in both samples across all scales (0.78 to 0.85). Validity: Criterion (significant mean differences between the community and clinical subsamples).
Objectified Body Consciousness Scales: Surveillance, Body Shame, and Control [43]	(16)	Fem	17–39	Col	Reliability: Acceptable with high internal consistency across the three scales (0.72 to 0.89). Validity: The scales were negatively correlated with “body esteem”, but the correlations were modest (−0.51) to low (−0.16).
Body Shape Questionnaire [44]	(14)	Fem	20–24	Com/Clin/Coll	Reliability: Not reported. Validity: Criterion (significant mean differences between the community and clinical subsamples) and convergent (high *r* with body dissatisfaction measure [0.66]).
Factor I of the Eating Attitudes Test [45]	(7)	Fem	18–25	Clin/Coll	Reliability: High internal consistency in both subsamples (0.86 to 0.90). Validity: Criterion (significant mean differences between the community and clinical subsamples) and convergent (high *r* with body image composite [0.61]).
Muscularity-Oriented (n = 14 depression studies, avg *r* = 0.23 [men] and 0.19 [women]; n = 5 anxiety studies, avg *r* = 0.23 [men] and 0.019 [women]; n = 6 self-esteem studies, avg *r* = −0.20 [men] and −0.15 [women])					
Drive for Muscularity Scale [24]	(51)	Male and Fem	18–24	HS	Reliability: High internal consistency (0.84). Validity: Modest convergence with Mental health indicators such as self-esteem (−0.41) and depression (0.32) but small and nonsignificant association with others (e.g., eating disorder symptoms (−0.05); body-image dissatisfaction (−0.15)).
Male Body Attitudes Scales: Muscularity, Low Body Fat, and Height [25]	(23)	Male	16–62	Col	Reliability: High internal consistency/test–retest reliability for the total (0.91/0.91) and the scales (0.88/0.83 to 0.93/0.94). Validity: Evidence of construct validity with many body-image related measures (0.40 to 0.91), but not always (0.13 to 0.33).
Body Esteem (Male) Scales: Physical Attractiveness, Upper Body Strength, Physical Condition [46]	(9)	Male and Fem	nr	Col	Reliability: Adequate to high internal consistency for the scales (0.81 to 0.86). Validity: The individual scales correlated substantively with self-esteem in males (0.45 to 0.51).
Muscle Dysmorphic Disorder Inventory and Scales: Desire for Size, Appearance Intolerance, and Functional Impairment [47]	(8)	Mal	18–72	Com/WL	Reliability: The test–retest reliability was high (0.87). Validity: Evidence of convergent validity with many body-image related measures (0.45 to 0.68), but not always (0.06 to 0.35).
Body Parts Satisfaction Scale for Males: Face, Legs, Upper Body [48]	(6)	Mal	18–22	Col	Reliability: The internal consistency (0.87 to 0.97) and test–retest reliability (0.58 to 0.94) were high across the scales. Validity: Evidence of convergent validity was mixed (−0.02 to 0.49).
Non-Specific concerns (n = 12 depression studies, avg *r* = 0.34 [men] and 0.29 [women]; n = 5 anxiety studies, avg *r* = −0.24 [men] and −0.27 [women]; n = 7 self-esteem studies, avg *r* = −0.55 [men] and −0.57 [women])					
Multidimensional Body Self-Relations Questionnaire [49]	(24)	Male and Fem	15–87	Com	Reliability: High internal consistency (0.94). Validity: Strong associations with related constructs (0.50 to 0.73).
Body Appreciation Scale [50]	(12)	Fem	18–22	Col	Reliability: High internal consistency in both subsamples (~0.94). Validity: The scores were positively correlated with appearance evaluation and negatively related to body dissatisfaction for women and men (0.80 to −76).

Notes: (n) = number of studies that administered the measure to >30 male participants; Gen = gender of the validating study; Age = age range of the sample; Pop = sampled population; Clin = Clinical; Col = college; Com = community; HS = high school; Com WLs = community weight lifters; nr = age not reported.

## Data Availability

Our data are available upon request from the corresponding author.

## References

[1] Ward Z.J., Rodriguez P., Wright D.R., Austin S.B., Long M.W. (2019). Estimation of Eating Disorders Prevalence by Age and Associations With Mortality in a Simulated Nationally Representative US Cohort. JAMA Netw. Open.

[2] Arcelus J., Mitchell A.J., Wales J., Nielsen S. (2011). Mortality rates in patients with anorexia nervosa and other eating disorders. A meta-analysis of 36 studies. Arch. Gen. Psychiatry.

[3] Blinder B.J., Cumella E.J., Sanathara V.A. (2006). Psychiatric comorbidities of female inpatients with eating disorders. Psychosom. Med..

[4] Milos G., Spindler A., Hepp U., Schnyder U. (2004). Suicide attempts and suicidal ideation: Links with psychiatric comorbidity in eating disorder subjects. Gen. Hosp. Psychiatry.

[5] Milos G.F., Spindler A.M., Buddeberg C., Crameri A. (2003). Axes I and II comorbidity and treatment experiences in eating disorder subjects. Psychother. Psychosom..

[6] Cash T.F. (2004). Body image: Past, present, and future. Body Image.

[7] Tiggemann M. (2004). Body image across the adult life span: Stability and change. Body Image.

[8] Slade P.D. (1994). What is body image?. Behav. Res. Ther..

[9] Polivy J., Herman C.P. (2002). Causes of eating disorders. Annu. Rev. Psychol..

[10] Phelps L., Johnston L.S., Augustyniak K. (1999). Prevention of eating disorders: Identification of predictor variables. Eat. Disord. J. Treat. Prev..

[11] Neumark-Sztainer D., Story M., Resnick M.D., Garwick A., Blum R.W. (1995). Body dissatisfaction and unhealthy weight-control practices among adolescents with and without chronic illness: A population-based study. Arch. Pediatr. Adolesc. Med..

[12] Sim L., Zeman J. (2006). The Contribution of Emotion Regulation to Body Dissatisfaction and Disordered Eating in Early Adolescent Girls. J. Youth Adolesc..

[13] van den Berg P.A., Mond J., Eisenberg M., Ackard D., Neumark-Sztainer D. (2010). The link between body dissatisfaction and self-esteem in adolescents: Similarities across gender, age, weight status, race/ethnicity, and socioeconomic status. J. Adolesc. Health.

[14] Caldwell M.B., Brownell K.D., Wilfley D.E. (1997). Relationship of weight, body dissatisfaction, and self-esteem in African American and white female dieters. Int. J. Eat. Disord..

[15] Goldfield G.S., Moore C., Henderson K., Buchholz A., Obeid N., Flament M.F. (2010). Body dissatisfaction, dietary restraint, depression, and weight status in adolescents. J. Sch. Health.

[16] Wiederman M.W., Pryor T.L. (2000). Body dissatisfaction, bulimia, and depression among women: The mediating role of drive for thinness. Int. J. Eat. Disord..

[17] Barnes M., Abhyankar P., Dimova E., Best C. (2020). Associations between body dissatisfaction and self-reported anxiety and depression in otherwise healthy men: A systematic review and meta-analysis. PLoS ONE.

[18] Cameron E.M., Ferraro F.R. (2004). Body satisfaction in college women after brief exposure to magazine images. Percept. Mot. Ski..

[19] Cash T.F., Henry P.E. (1995). Women’s body images: The results of a national survey in the U.S.A. Sex Roles.

[20] Bonafini B.A., Pozzilli P. (2011). Body weight and beauty: The changing face of the ideal female body weight. Obes. Rev..

[21] (2023). Gerbner’s Cultivation Theory in Media Communication. https://www.simplypsychology.org/cultivation-theory.html.

[22] Thompson J.K. (2004). The (mis)measurement of body image: Ten strategies to improve assessment for applied and research purposes. Body Image.

[23] Grogan S. (2010). Promoting Positive Body Image in Males and Females: Contemporary Issues and Future Directions. Sex Roles.

[24] McCreary D.R., Sasse D.K. (2000). An exploration of the drive for muscularity in adolescent boys and girls. J. Am. Coll. Health.

[25] Tylka T.L., Bergeron D., Schwartz J.P. (2005). Development and psychometric evaluation of the Male Body Attitudes Scale (MBAS). Body Image.

[26] Gleaves D.H., Cepeda-Benito A., Williams T.L., Cororve M.B., Fernandez M.d.C., Vila J. (2000). Body image preferences of self and others: A comparison of spanish and american male and female college students. Eat. Disord..

[27] Cohn L.D., Adler N.E. (1992). Female and male perceptions of ideal body shapes: Distorted views among Caucasian college students. Psychol. Women Q..

[28] Connor-Greene P.A. (1988). Gender differences in body weight perception and weight-loss strategies of college students. Women Health.

[29] Schooler D., Ward L.M. (2006). Average Joes: Men’s relationships with media, real bodies, and sexuality. Psychol. Men Masculinity.

[30] Leit R.A., Pope H.G., Gray J.J. (2001). Cultural expectations of muscularity in men: The evolution of playgirl centerfolds. Int. J. Eat. Disord..

[31] Pope H.G., Olivardia R., Gruber A., Borowiecki J. (1999). Evolving ideals of male body image as seen through action toys. Int. J. Eat. Disord..

[32] Frederick D.A., Buchanan G.M., Sadehgi-Azar L., Peplau L.A., Haselton M.G., Berezovskaya A., Lipinski R.E. (2007). Desiring the muscular ideal: Men’s body satisfaction in the United States, Ukraine, and Ghana. Psychol. Men Masculinity.

[33] Strother E., Lemberg R., Stanford S.C., Turberville D. (2012). Eating Disorders in Men: Underdiagnosed, Undertreated, and Misunderstood. Eat. Disord..

[34] Jankowski G.S., Diedrichs P.C., Halliwell E. (2014). Can appearance conversations explain differences between gay and heterosexual men’s body dissatisfaction?. Psychol. Men Masculinity.

[35] Shunmuga Sundaram C., Dhillon H.M., Butow P.N., Sundaresan P., Rutherford C. (2019). A systematic review of body image measures for people diagnosed with head and neck cancer (HNC). Support. Care Cancer.

[36] Gardner R.M., Brown D.L. (2010). Body image assessment: A review of figural drawing scales. Personal. Individ. Differ..

[37] Kling J., Kwakkenbos L., Diedrichs P.C., Rumsey N., Frisén A., Brandão M.P., Silva A.G., Dooley B., Rodgers R.F., Fitzgerald A. (2019). Systematic review of body image measures. Body Image.

[38] Edwards C., Tod D., Molnar G. (2014). A systematic review of the drive for muscularity research area. Int. Rev. Sport Exerc. Psychol..

[39] Anderson C.B., Bulik C.M. (2004). Gender differences in compensatory behaviors, weight and shape salience, and drive for thinness. Eat. Behav..

[40] Arksey H., O’Malley L. (2005). Scoping studies: Towards a methodological framework. Int. J. Soc. Res. Methodol..

[41] Garner D.M., Olmstead M.P., Polivy J. (1983). Development and validation of a multidimensional eating disorder inventory for anorexia nervosa and bulimia. Int. J. Eat. Disord..

[42] Fairburn C.G., Beglin S.J. (1994). Assessment of eating disorders: Interview or self-report questionnaire?. Int. J. Eat. Disord..

[43] McKinley N.M., Hyde J.S. (1996). The Objectified Body Consciousness Scale: Development and Validation. Psychol. Women Q..

[44] Cooper P.J., Taylor M.J., Cooper Z., Fairburn C.G. (1987). The development and validation of the Body Shape Questionnaire. Int. J. Eat. Disord..

[45] Garner D.M., Olmsted M.P., Bohr Y., Garfinkel P.E. (1982). The Eating Attitudes Test: Psychometric features and clinical correlates. Psychol. Med..

[46] Franzoi S.L., Shields S.A. (1984). The Body Esteem Scale: Multidimensional Structure and Sex Differences in a College Population. J. Personal. Assess..

[47] Hildebrandt T., Langenbucher J., Schlundt D.G. (2004). Muscularity concerns among men: Development of attitudinal and perceptual measures. Body Image.

[48] McFarland M.B., Petrie T.A. (2012). Male body satisfaction: Factorial and construct validity of the Body Parts Satisfaction Scale for men. J. Couns. Psychol..

[49] Brown T.A., Cash T.F., Mikulka P.J. (1990). Attitudinal Body-Image Assessment: Factor Analysis of the Body-Self Relations Questionnaire. J. Personal. Assess..

[50] Avalos L., Tylka T.L., Wood-Barcalow N. (2005). The Body Appreciation Scale: Development and psychometric evaluation. Body Image.

